# Apolipoprotein ɛ4 breaks the association between declarative long-term memory and memory-based orienting of spatial attention in middle-aged individuals

**DOI:** 10.1016/j.cortex.2016.06.002

**Published:** 2016-09

**Authors:** Gerardo Salvato, Eva Z. Patai, Tayla McCloud, Anna C. Nobre

**Affiliations:** a Oxford Centre for Human Brain Activity, University of Oxford, Oxford, United Kingdom; b Department of Experimental Psychology, University of Oxford, Oxford, United Kingdom; c Department of Brain and Behavioural Sciences, University of Pavia, Pavia, Italy; d Cognitive Neuropsychology Centre, Niguarda Ca' Granda Hospital, Milano, Italy

**Keywords:** APOE, Memory-based attention, Declarative memory, Spatial attention, Alzheimer disease

## Abstract

Apolipoprotein (APOE) ɛ4 genotype has been identified as a risk factor for late-onset Alzheimer disease (AD). The memory system is mostly involved in AD, and memory deficits represent its key feature. A growing body of studies has focused on the earlier identification of cognitive dysfunctions in younger and older APOE ɛ4 carriers, but investigation on middle-aged individuals remains rare. Here we sought to investigate if the APOE ɛ4 genotype modulates declarative memory and its influences on perception in the middle of the life span. We tested 60 middle-aged individuals recruited according to their APOE allele variants (ɛ3/ɛ3, ɛ3/ɛ4, ɛ4/ɛ4) on a long-term memory-based orienting of attention task. Results showed that the APOE ɛ4 genotype impaired neither explicit memory nor memory-based orienting of spatial attention. Interestingly, however, we found that the possession of the ɛ4 allele broke the relationship between declarative long-term memory and memory-guided orienting of visuo-spatial attention, suggesting an earlier modulation exerted by pure genetic characteristics on cognition. These findings are discussed in light of possible accelerated brain ageing in middle-aged ɛ4-carriers, and earlier structural changes in the brain occurring at this stage of the lifespan.

## 1 Introduction

Dementia is a neurodegenerative disorder affecting at about 10% of the worldwide ageing population (Subramaniam et al., 2015). One of the main challenges for neuroscientists is to identify early cognitive markers before its clinical manifestation. Although the pathogenesis of dementia is still unclear, a number of possible risk factors have been identified (Knopman, Mosley, Catellier, & Coker, 2009). Pure genetic characteristics, such as the possession of the Apolipoprotein ɛ4 allele (APOE ɛ4) may increase the odds for developing this disease (Albert et al., 2014, Wisdom et al., 2011). A growing body of studies has focused on the APOE ɛ4 effect over the lifespan in healthy individuals at risk for developing dementia. According to the gene-dose effect hypothesis, inheritance of one ɛ4 allele is associated with increased risk of late-onset Alzheimer's disease (AD) in older adults. If two ɛ4 alleles are inherited the risk is increased further (Corder et al., 1993). So far, however, evidence is mixed as to the effects of APOE ɛ4 on cognition, and this genotype seems to exert a different modulation on functions depending on age (Marchant et al., 2010, Small et al., 2004, Wisdom et al., 2011). The possibility of an antagonistic pleiotropy has been suggested, whereby possession of APOE ɛ4 alleles confers cognitive advantage in younger participants though this reverts to cognitive decline with advancing age (Tuminello & Han, 2011).

It is currently unclear at which stage of the lifespan the APOE ɛ4 allele may start to exert a disadvantageous effect on cognition. If APOE ɛ4 genotype results in increasing cognitive deficits over the life span, then middle-aged individuals might be expected to show cognitive decline on sensitive tasks. Behavioural studies on middle-aged individuals have shown the full gamut of results. Positive (Evans et al., 2014, Jochemsen et al., 2012), negative (Flory et al., 2000, Jonaitis et al., 2013), and null (Bunce et al., 2014, Nilsson et al., 2006, Sager et al., 2005) effects of ɛ4 allele on cognitive function have been reported in this age group (see Salvato, 2015 for a recent review). Mixed findings have also emerged from neuroimaging studies looking at functional activations during memory tasks, in which ɛ4-carriers exhibited paradoxical engagement of temporal, parietal and frontal areas traditionally associated with memory (Evans et al., 2014, Filippini et al., 2011, Trachtenberg et al., 2012).

The relationship between the APOE genotype and the declarative memory system in middle-aged individuals needs to be further clarified, as impairments in learning and retrieving new information represent key features of dementia (Grober and Buschke, 1987, Grober et al., 1988, Grossman et al., 2006). Furthermore, it is increasingly acknowledged that long-term memory also plays an important role in facilitating perception by modulating attention to relevant locations (Bar, 2009, Chun and Johnson, 2011, Hutchinson and Turk-Browne, 2012, Nobre and Mesulam, 2014, Summerfield et al., 2006). Our laboratory has shown, for example, that spatial attention is tuned according to memory traces of previous target locations within given environments, with neural modulation occurring from early stages of visual perception (Stokes et al., 2012, Summerfield et al., 2011). This mechanism is preserved in healthy older individuals, despite significant impairments in explicit memory for spatial and contextual associations (Salvato, Patai, & Nobre, 2015). Therefore, one important question is whether individuals at risk for developing dementia may suffer from impairment in memory-based orienting of attention, and whether subtle deficits may influence perception and cognition even decades before the clinical manifestation of dementia.

In our study, we sought to provide further evidence about APOE risk factors and spatial associative memory in middle age. We asked whether individuals at-risk for dementia show memory impairments, and if so, whether this diminishes their ability to utilize spatial contextual memory to facilitate perception. To this aim we tested sixty healthy middle-aged individuals on a task developed in our lab, which is designed to test memory-guided attention, using naturalistic stimuli. We compared performance on this task across three genotypic groups: ɛ3/ɛ3, ɛ3/ɛ4, ɛ4/ɛ4. We predicted that, if the ɛ4 allele is indeed a risk factor for dementia and associated cognitive deficits, specifically in the memory domain, and then we would find an effect of ɛ4 dosage in middle age. If memory impairments were present, we might expect downstream deficits in memory-based orienting of attention. Results showed that APOE genotype did not modulate explicit memory or attention performance per se. However, we found that the ɛ4 allelic variant broke the association typically observed between memory-based orienting of attention and the declarative contextual memory, suggesting that alternative memory systems or mechanisms guide attention in their case.

## 2 Materials and methods

### 2.1 Participants

Sixty healthy middle-aged individuals participated in the study. Potential participants were invited according to their APOE allelic variants (ɛ3/ɛ3, ɛ3/ɛ4, ɛ4/ɛ4), through the Oxford Biobank, an age-stratified random sample of 1800 healthy men and women from Oxfordshire. Twenty individuals were recruited for each of the three APOE allelic variants. Inclusion criteria consisted of absence of referred neurological or psychiatric diseases, and preservation of general cognitive functioning, measured by means of a neuropsychological screening. All participants were native English speakers, and had normal or corrected-to-normal vision. Informed consent was obtained prior to participation in the experiment according to the Declaration of Helsinki. The experimental protocol had ethical approval from the University of Oxford Central University Research Ethics Committee. Participants were remunerated £30 for their time.

### 2.2 Cognitive assessment

The Addenbrooke's Cognitive Examination–third version (ACE-III) (Hsieh, Schubert, Hoon, Mioshi, & Hodges, 2013) was administered to the participants in order to rule out any cognitive deficits. The ACE is a brief neuropsychological screening battery, widely adopted in the clinical practice to detect cognitive decline associated with dementias, such as AD. The ACE-III has been recently identified as the best alternative to the Mini-Mental State Examination (MMSE) (Folstein, Robins, & Helzer, 1983) in detecting dementia (Tsoi, Chan, Hirai, Wong, & Kwok, 2015). It assesses five main cognitive domains: attention, memory, fluency, language, and visuo-spatial abilities. The total score of ACE-III is 100, and a performance below 88 indicates a suspicion of dementia.

### 2.3 Apolipoprotein E genotyping

APOE genotyping was carried out by the Oxford Biobank, using Applied Bio-systems, Assay-on-demand TaqMan^®^ SNP Genotyping As-says, C_3084793_20 and C_904973_10 corresponding to APOE SNPs rs429358 and rs7412, respectively, and run on an ABI 7900HT Fast Real-Time PCR system. Haplotypes corresponding to APOE ɛ3 and ɛ4 were then deduced. Genetic information was not disclosed to the participants and examiners who performed the testing.

## 3 Task and procedure

### 3.1 Stimuli

The task consisted of three phases in which participants: (1) learnt spatial contextual associations of target objects embedded within complex scenes, (2) performed a target detection task in which the learnt spatial contextual associations could provide memory-based cues to guide performance, and (3) completed explicit recollection and recognition judgements about identity and location of the target objects in their associated scenes. The task design, materials, and procedures were the same as those used in our previous study investigating memory-based attention in ageing (Salvato et al., 2015).

The contextual memories were created using complex scenes of indoor and outdoor scenes in which a target object was positioned randomly. Ninety-six colour photographs of indoor and outdoor scenes were selected from the Flickr Creative Commons (Judd, Ehinger, Durand, & Torralba, 2009). The objects were obtained from the SUN dataset (Xiao, Hays, Ehinger, Oliva, & Torralba, 2010). They were fit into a 100 × 100 pixels transparent box (3.4° × 4.5°) when superimposed on the scene, and a 150 × 150 pixels transparent box (5.2° × 6.7°) when presented against a grey background. Object placement did not necessarily mirror realistic positioning within its context, and objects were not necessarily semantically related to its associated scene. The placement of objects within scenes, both in terms of scene-pairing and location, was counterbalanced across experimental conditions over participants. Eye movements were recorded using an eye-tracking camera (Eye-link, SR Research) during all phases of the experiment.

#### 3.1.1 Learning

During the first phase, participants performed a learning task in which they were asked to find the target object in each of the scenes. At the beginning of each trial, the object was presented centrally against a grey background for three seconds. This was followed immediately by the presentation of a scene in which the search target was embedded. Once participants had found the object on the scene, they clicked on the left mouse button and a mouse cursor appeared at the centre of the screen. They then moved the cursor on the object and clicked again. They received feedback “object found!” or “object not found!” according to their search performance. Mouse clicks falling outside a 50-pixel diameter circle around the target location were considered as errors. Participants completed four learning blocks separated by rest breaks. In each block, each of the 96 scenes was presented with the same associated object at the same spatial location. At the end of each block, the total number of objects found was displayed. Scene order was randomised across blocks. The learning task lasted approximately 45 min in total.

At the end of the learning phase, participants had a 30-min rest during which they were comfortably seated in a different room, and engaged in conversation with the experimenter. In order to avoid any interference with the contextual memory consolidation, the use of any devices or printed materials involving picture of scenes or objects was avoided.

#### 3.1.2 Memory-based orienting of attention

After the long break, participants performed the memory-guided attention task. Seventy-two of the 96 studied scenes were used in this phase. Participants were asked to fix their gaze on a cross appearing on the centre of the monitor during the whole task. At the beginning of each trial, a fixation-cross appeared on the screen warning participants that a scene was about to appear (1000–1500 msec randomised interval). One of the previously studied scenes then appeared. On the majority of the trials (89%), the target object associated with that scene flashed briefly (onset jittered between 1000 and 1500 msec after scene onset for a duration of 100 msec) either at the learnt location (valid memory cues, 50% of trials) or at a different location (of the opposite hemifield, 50% invalid memory cues). These memory-based cues were highly predictive of the target location, since when the target did not occur at the remembered location, it could occur in any other location within the other side of the scene. Therefore, the probability that the object occurred at any other, uncued location was much lower. On a minority of trials, a hexagonal stop sign appeared instead. Participants were required to detect the presence of the stimuli on the scene. They were instructed to click the left mouse button if the target appeared and to refrain from responding if the hexagonal foil stimulus appeared. Altogether there were 32 valid-cue trials, 32 invalid-cue trials, and 8 foil trials. Object locations were counterbalanced across participants, so that objects were equally likely to occur in each hemifield, and in valid and invalid cueing conditions.

#### 3.1.3 Explicit retrieval

In the last phase, participants were tested on their explicit memory for the spatial contextual associations. At the beginning of each trial, they viewed a scene without its target object. They were required to indicate the spatial location in which the object appeared during the learning phase. Once participants retrieved the location of the remembered target, they clicked the left mouse button. A white cursor appeared at the centre of the screen, which participants then moved to the exact remembered location. They had to locate the cursor within a 2-min time window. This allowed for ample time for most participants to locate the object on the majority of trials. Following cursor placement, the scene disappeared, and they rated their confidence level for memory for the object location, pressing the left (1 = “not at all confident”), middle (2 = “fairly confident”), or right (3 = “very confident”) mouse button. After a blank fixation period (1000–15,000 msec), three objects appeared, and participants indicated which item was previously associated with the scene during the learning phase, in a three-alternative forced-choice recognition (3AFC) task. The objects were aligned horizontally (each within a transparent box of 150 × 150 pixels) and participants used the left, middle, or right mouse button indicating the object at left, middle or right position on the screen respectively. Following the response, they again rated their confidence level in the memory for object identity. During this phase, participants were free to move their eyes.

#### 3.1.4 Apparatus

The tasks were programmed using Presentation (Neurobehavioural Systems, Albany, NY). A personal computer controlled the stimulus displays and collected the responses. The stimuli were displayed on a 24-inch monitor with a resolution of 1028 by 768 pixels and a 60-Hz refresh rate.

## 4 Results

### 4.1 Final sample

One participant in the ɛ4/ɛ4 group scored below our cut-off (<86) for the ACE-III screening. Data from this participant were therefore not included in the analysis. The different APOE genotype groups were matched for age [*F*(2, 78) = .6, *p* = .552], gender [χ^2^(2, *N* = 59) = 2.2; *p* = .336], level of education [*F*(2, 78) = .07, *p* = .931], family history of dementia [χ^2^(2, *N* = 59) = .1; *p* = .923], handedness [χ^2^(2, *N* = 59) = 2.2; *p* = .329], and ACE-III scores [*F*(2, 78) = .3, *p* = .737]. Demographic characteristics according to APOE genotype are presented in Table 1.

### 4.2 Learning

**Fig. 1:**
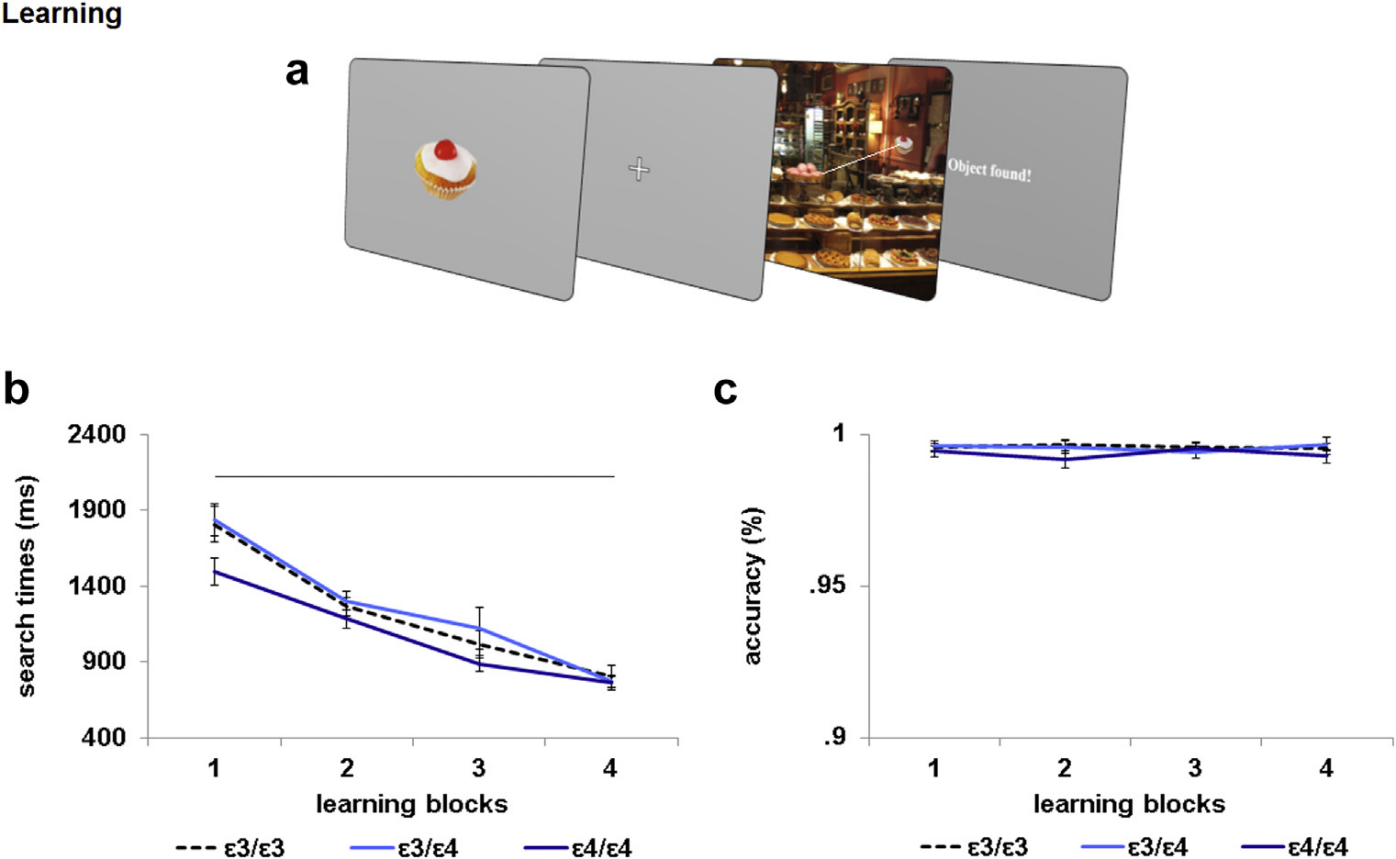
Phase 1: Learning task and results. (a) Schematic illustration of the task structure. An object was presented for 3 sec at the centre of the screen. Soon after, a scene containing the target appears. Participants were instructed to find the target. (b) The three APOE groups showed the decrease in Search Times over the learning session. (c) Participants were at ceiling effect in finding the target objects on the scenes, irrespective to the APOE Genotype.

Learning performance among the three groups was compared using the percentage of targets found (Search Accuracy) and the mean time to the first mouse click (Search Times) over the four learning blocks. We found no difference between the APOE genotype groups comparing accuracy and search times. Nevertheless, results showed that APOE ɛ4/ɛ4 improved their learning over the four blocks with a slower rate compared to the other groups. A mixed ANOVA was performed with Search Accuracy across different Blocks (1, 2, 3, 4) as a within-subject factor and APOE Genotype (ɛ3/ɛ3, ɛ3/ɛ4, ɛ4/ɛ4) as a between-subjects factor. Performance across participants was highly accurate from the first learning block. There was no accuracy improvement over Blocks [*F* = (3, 56) = .10, *p* = .958; η^2^_*p*_ = .002] or differences among Genotype [*F* = (2, 56) = 1.19, *p* = .310; η^2^_*p*_ = .041]. The interaction of Blocks by APOE Genotype was also far from significant [*F* = (6, 56) = .72, *p* = .632; η^2^_*p*_ = .025 (Fig. 1).

An equivalent ANOVA using Search Times showed that participants became faster in finding the target objects over the learning blocks [linear contrast of block: *F* = (1, 56) = 241.31, *p* < .001; η^2^_*p*_ = .812]. There was no significant effect of APOE genotype [*F* = (2, 56) = 2.1, *p* = .132; η^2^_*p*_ = .070] or interaction between Genotype and the linear contrast of Block [*F* = (2, 56) = 2.04, *p* = .140; η^2^_*p*_ = .068 (Fig. 1).

To explore the learning performance further we used a more sensitive measure, comparing changes in search-time slopes between groups over the four blocks. We calculated the linear regression line (Search Times Slopes) through data points in Search Times and in learning Blocks for each participant. We performed a Univariate ANOVA with Search-Time Slopes as a dependent variable and Genotype as a between-subjects factor. Results revealed a main effect of Genotype. Post-hoc Bonferroni-adjusted multiple comparisons showed that the ɛ4/ɛ4 (*M* = −.005; *SE* = 0) group learnt at a slower rate compared to the ɛ3/ɛ4 (*M* = −.003; *SE* = 0) (*p* = .021) and ɛ3/ɛ3 (*M* = −.003; *SE* = 0) (*p* = .027) group. These results may reflect the fact that ɛ4/ɛ4 individuals were faster on the first learning block compared to other groups. We then performed a one-way ANOVA with Search Times of block 1 as dependent variable and Genotype as between-subject factor. We found a main effect of Genotype [*F* = (2, 58) = 3.24, *p* = .046; η^2^_*p*_ = .104]. Post-hoc Bonferroni-adjusted multiple comparisons revealed a statistical trend in favour of ɛ4/ɛ4 (*M* = 1493; *SE* = 106) being faster than ɛ3/ɛ4 (*M* = 1838; *SE* = 103) (*p* = .073). There was no difference between ɛ4/ɛ4 and ɛ3/ɛ3 (*M* = 1806; *SE* = 103) (*p* = .121) or between ɛ3/ɛ3 and ɛ3/ɛ4 (*p* > .05). These findings demonstrated that ɛ4/ɛ4 participants were faster than others group at the beginning of the leaning phase but then had shallower learning slopes.

### 4.3 Memory-based orienting of attention

All participants were able to inhibit their response to the foil stimulus. The false-alarm rate was calculated using foil trials, namely when the stop sign appeared instead of the target object associated with a particular scene. We found no difference between Genotype groups [*F* = (2, 58) = .53, *p* = .588; η^2^_*p*_ = .019] (ɛ3/ɛ3: *M* = .18, *SE* = .03; ɛ3/ɛ4: *M* = .14, *SE* = .03; ɛ4/ɛ4: *M* = .14, *SE* = .03).

In order to explore the orienting effect, we used the 64 scenes excluding trials with foils. This set of scenes contained an equal number of trials with targets occurring in valid (32 trials) and invalid (32 trials) locations. Reaction times to detect the target objects provided the main dependent variable for testing the effects of memory-based orienting of attention. Scenes in which participants had not located the target objects in the third and the forth blocks during the learning were excluded from the analysis. Trials in which RTs were above or below 2 standard deviations were also excluded. The average percentage of trials discarded in each of the three groups (ɛ3/ɛ3: *M* = 4.6%, *SE* = .31; ɛ3/ɛ4: *M* = 5.3%, *SE* = .31; ɛ4/ɛ4: *M* = 4.7%, *SE* = .32).

**Fig. 2:**
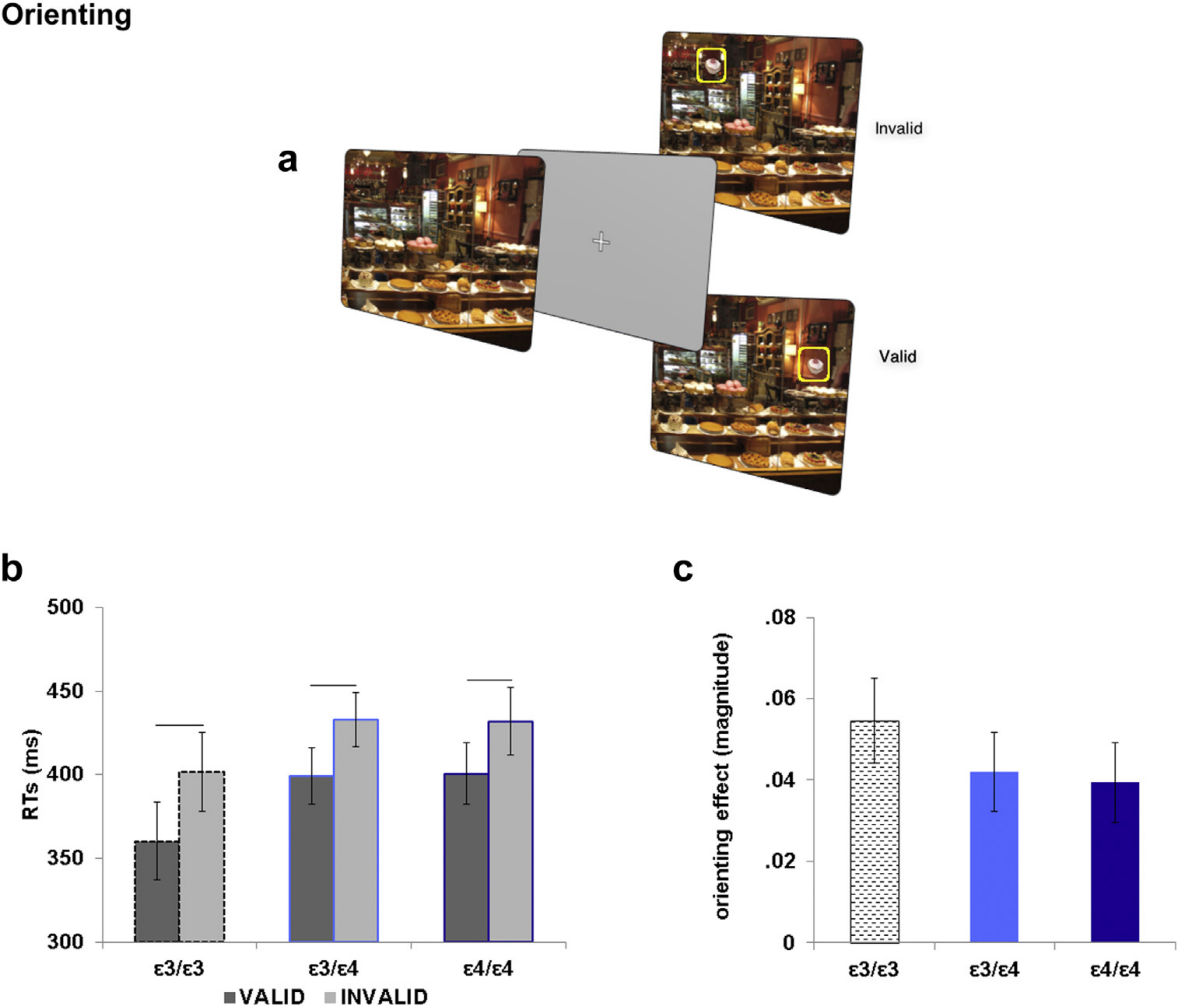
Orienting: experimental task and results. (a) Schematic illustration of the task structure. At the beginning of each trial, a cue scene previously associated with a target. After a variable amount of time (1000–1500 msec), the target object appeared for 100 msec in the previously learnt location (valid trials, here indicated in a yellow square at bottom row) or in a new spatial position (invalid trials, here indicated in a yellow square at top row). Participants were instructed to press a mouse button as soon as they see the target object appearing on its scene. (b) Mean RTs revealed that the three APOE groups showed a reliable significant difference between valid and invalid trials. (c) The magnitude of the orienting effect was equal between the three groups.

A mixed ANOVA tested for the effects of Validity (valid, invalid) as a within-subjects factor, and Genotype (ɛ3/ɛ3, ɛ3/ɛ4, ɛ4/ɛ4) as a between-subjects factor. A reliable main effect of Validity was observed [*F* = (1, 56) = 58.63, *p* < .001; η^2^_*p*_ = .511] in the absence of any main effect of Genotype [*F* = (2, 56) = 1.35, *p* = .267; η^2^_*p*_ = .046] or interaction [*F* = (2, 56) = .44, *p* = .637; η^2^_*p*_ = .016]. Overall, all groups benefited from the appearance of the target object in a previously learnt location on the scene (Fig. 2).

We also tested for possible differences in orienting between Genotype groups by comparing the magnitude of the orienting effects, using the formula: [(invalid – valid)/(invalid + valid)], a measure that normalized the effect based on RT (see Salvato et al., 2015). A one-way ANOVA with Orienting Effect as dependent variable and Genotype as fixed factor showed no main effect of Genotype [*F* = (2, 58) = .67, *p* = .514; η^2^_*p*_ = .023]. A reliable orienting effect occurred in the three APOE groups (ɛ3/ɛ3: *M* = .05; *SE* = .01; ɛ3/ɛ4: *M* = .04; *SE* = .01; ɛ4/ɛ4: *M* = .04; *SE* = .01) (Fig. 2).

To supplement the frequentist statistical analyses performed on the orienting effect, a Bayesian one-way ANOVA (Love et al., 2015) was used to test whether there was evidence for supporting the null hypothesis against the alternative hypothesis. The Bayes Factor (BF_10_) provides an odds ratio for the alternative/null hypotheses (values <1 favour the null hypothesis, and values >1 favour the alternative hypothesis). For example, a BF_10_ of .20 would indicate that the null hypothesis is 5 times (1:.20) more likely than the alternative hypothesis (see Jarosz & Wiley, 2014). Results supported the null against the alternative hypothesis with a *BF*_*10*_ = .22.

### 4.4 Explicit memory

Two measures during the explicit memory phase were used to assess the quality of the contextual associations for target location and target identity within the scenes, respectively: the distance between cursor placement and the actual position of the object in the studied scene and the mean accuracy in the 3AFC for selecting the correct identity of the object in the studied scene. Confidence ratings associated with these two measures were also analysed to explore participants' insight into their memory performance. Results indicated equivalent memory retrieval skills between APOE Genotype groups. Furthermore, all participants showed an equally strong congruence between the confidence ratings and the actual explicit memory scores, implying a certain awareness, or meta-memory, of performance.

**Fig. 3:**
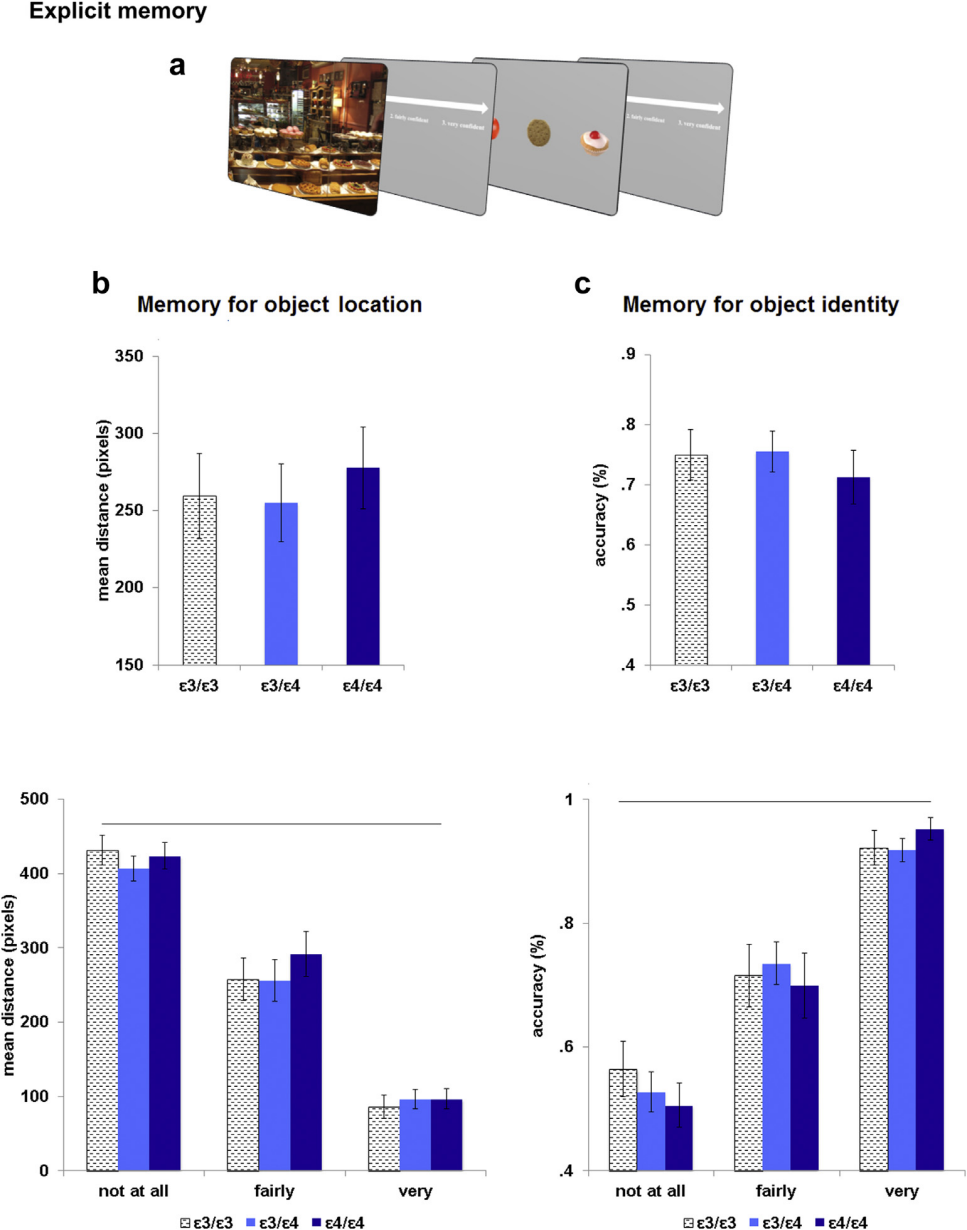
Memory session: experimental task and results. (a) Schematic illustration of the explicit memory task. At the beginning of each trial, a scene appeared on the screen. Participants were instructed to place the mouse cursor on the exact spatial location of the object associated with a specific scene (explicit memory for object location). They were also required to rate the confidence for their performance. Soon after, tree objects appeared on the screen and participants were required to choose the object associated with that scene (explicit memory for object identity). Lastly, they rated their level of confidence. (b) Results of memory for object location. Results revealed no difference between groups. Furthermore, the awareness for the memory performance increased as the reported spatial location was closer to the veridical one. (c) Results of memory for object identity. The APOE Genotype groups were equally accurate n reporting the correct object associated with its scene in the 3AFC task. Participants were more accurate as a function of their awareness for the memory performance.

A one-way ANOVA comparing Mean Distances from the actual object location among the APOE Genotype showed no differences in the explicit retrieval of the spatial memory [*F* = (2, 58) = .20, *p* = .814; η^2^_*p*_ = .007]. Genotype Groups also did not differ when Confidence Ratings were included as an additional within-subjects factor. Whereas distance measures decreased with increasing confidence ratings [linear contrast of Confidence Rating: *F*(1, 56) = 637.94; *p* < .001; η^2^_*p*_ = .919], there was no main effect of Genotype [*F*(2, 56) = .36; *p* = .697; η^2^_*p*_ = .013] or interaction between genotype and Confidence Rating [*F*(2, 56) = .16; *p* = .849; η^2^_*p*_ = .006] (Fig. 3).

Equivalent analyses of object-identity memory also yielded no Genotype effects. The one-way ANOVA comparing accuracy in the 3AFC among groups showed no effect of Genotype [*F*(2, 58) = .32; *p* = .727; η^2^_*p*_ = .001]. A mixed ANOVA including Confidence Ratings showed a significant effect of Confidence Rating [linear contrast of Confidence Rating: *F*(1, 56) = 276.90; *p* < .000; η^2^_*p*_ = .832], but no main effect of Genotype [*F*(2, 56) = .02; *p* = .981; η^2^_*p*_ = .001] or interaction between Genotype and Confidence Rating [*F*(2, 56) = .62; *p* = .541; η^2^_*p*_ = .022]. The effect of Confidence Rating indicated that participants had insight into their memory performance; they were more confident when they were more accurate (Fig. 3).

It is important to note that during the orienting phase, the target objects appeared at wrong locations in the invalid trials, and this condition might have interfered with explicit memory performance. In our previous study (Salvato et al., 2015), we checked for possible influence on memory caused by the orienting task, and we found no difference between those subsets of trials. Nevertheless, we double-checked for this possibility again, since in principle there might have been a difference in how different genotypes could influence how interference during an invalid trial could affect explicit memory. According to our task design, we were able to compare the memory performance between scenes that appeared during the learning and explicit memory tasks but not in the orienting (24 trials), to those that appeared during the orienting task as well (72 trials). The former could be considered as a pure memory measure, while the latter trials could have been influenced by invalid location in the orienting. A series of paired-samples *t*-tests within each group revealed no difference in memory for object location between the two sets of scenes (24 *vs* 72 trials) as assessed by the mean distance in pixels from the veridical location of the target [ɛ3/ɛ3: [*t*(19) = 1.10; *p* = .285]; ɛ3/ɛ4: [*t*(19) = .88; *p* = .389]; ɛ4/ɛ4: [*t*(18) = 1.20; *p* = .244]]. The same results were obtained in the case of memory for the object identity. We found no difference between the memory for object identity when using 24 versus 72 trials, as assessed by the accuracy for selecting the scene-associated target [ɛ3/ɛ3: [*t*(19) = 1.29; *p* = .210]; ɛ3/ɛ4: [*t*(19) = −.17; *p* = .816]; ɛ4/ɛ4: [*t*(18) = .16; *p* = .869]].

### 4.5 Relationship between explicit memory and orienting

In a final set of analyses, we tested the degree to which explicit memory performance correlated with the memory-based attention orienting effects in each group. As before, the orienting effect was calculated on 64 scenes (32 valid, 32 invalid), excluding foil trials. For measures of explicit memory, we used the same 64 scenes from which orienting effects were derived. We calculated the mean distance from the actual object location (memory for object location) and the accuracy of the 3AFC task (memory for object identity) based on the explicit retrieval performance achieved on these 64 scenes. We used non-parametric Spearman's rho in correlation analyses, which circumvents possible issues with correlation outliers. We implemented correlation *p*-values in each group using a Bayesian approach (Love et al., 2015). The Fisher *r*-to-*z* transformation was used to compare correlation coefficients between groups (Cohen, Cohen, West, & Aiken, 2013).

**Fig. 4:**
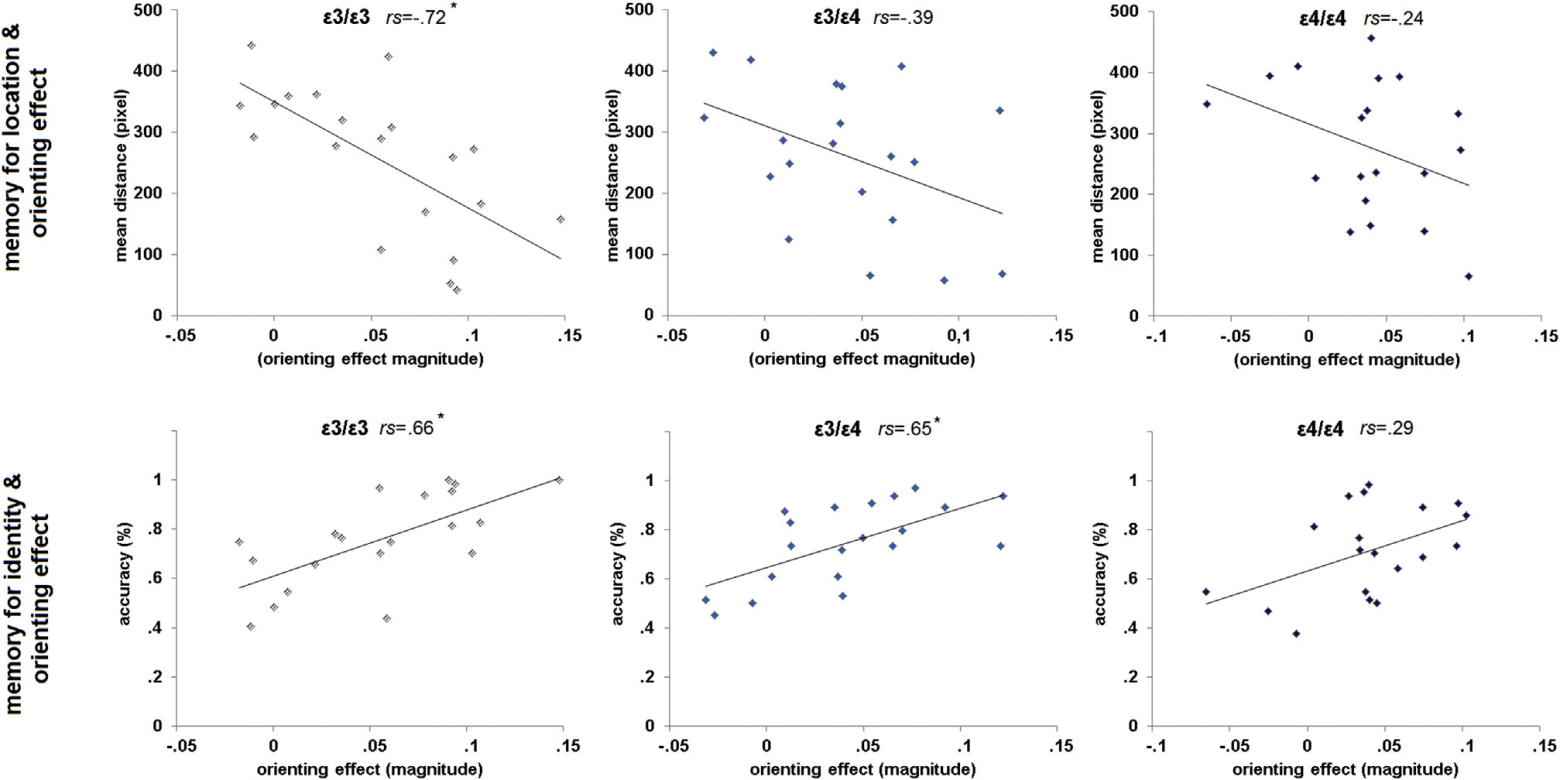
Relationship between the orienting effect and explicit memory for object location (top panels), or object identity (bottom panels). The graphs show the correlation between the magnitude of the orienting effect and the mean distance from the actual object location (upper panels), and between the orienting effect and the object identity accuracy (lower panels). While in the ɛ3/ɛ3 groups the orienting strongly relied on the explicit memory, this correlation dissipated as the ɛ4 allele dose increased. Results showed a lack of correlation between orienting effect and both memory for object location and identity.

We first looked at the correlation between explicit memory for object location and the orienting effect in each Genotype group. In the ɛ3/ɛ3 group we found a strong significant correlation between orienting effect and memory for the object location [*r*_*s*_(19) = −.72; *p* < .001; BF_10_ = 41.9 (very strong evidence for the alternative hypothesis)]. This correlation was weaker or absent in the ɛ4-carrier groups. While in the ɛ3/ɛ4 group we observed a statistical trend [*r*_*s*_(19) = −.39; *p* = .086; BF_10_ = 1.4 (anecdotal evidence for the alternative hypothesis)], in the ɛ4/ɛ4 participants the relationship between explicit retrieval of object location and memory-based orienting of attention was absent [*r*_*s*_(18) = −.24; *p* = .325; BF_10_ = .9 (anecdotal evidence for the null hypothesis) (Fig. 4). The present results are suggestive of a gene-dose effect of the ɛ4 allele (Corder et al., 1993), influencing the correlation between orienting of attention and explicit memory. Indeed, the magnitude of correlation coefficients appears to be inversely proportionate to the possession of ɛ4 alleles. To test for this effect, we performed a series of Helmert planned contrasts in which each category (except the last) is compared to the mean effect (averaged variable) of all subsequent categories. We compared correlation coefficients of ɛ3/ɛ3 (*r*_s_ = −.72) versus the mean of the other groups (ɛ3/ɛ4 + ɛ4/ɛ4/2) (*r*_s_ = −.31), and ɛ3/ɛ4 (*r*_s_ = −.39) versus ɛ4/ɛ4 (*r*_s_ = −.24). Helmert comparisons revealed that the possession of ɛ4 allele modulates the correlation between orienting and memory for object location compared to the non-carriers (*z* = −2; *p* = .04). Furthermore, having two ɛ4 alleles compared to one ɛ4 allele does not influence the correlations (*z* = −.4; *p* = .631). These results demonstrated a lack of gene-dose effect, although carrying the ɛ4 allele impairs the relationship between spatial explicit memory and orienting of spatial attention.

We also examined the correlation between memory for object identity and orienting effect in each Genotype group. The ɛ3/ɛ3 group showed significant correlation between the item memory and the memory-based orienting of attention [*r*_*s*_(19) = .66; *p* = .002; BF_10_ = 36.9 (very strong evidence for the alternative hypothesis)]. The same result was observed in ɛ3/ɛ4 participants [*r*_*s*_(19) = .65; *p* = .002; BF_10_ = 22.2 (strong evidence for the alternative hypothesis)] but not in the ɛ4/ɛ4 individuals, for which the correlation was absent [*r*_*s*_(18) = .29; *p* = .229; BF_10_ = 2.0 (anecdotal evidence for the alternative hypothesis)]. As before, we performed planned comparisons on correlation coefficients [ɛ3/ɛ3 (*r*_s_ = .66) *vs* the mean of the other groups (ɛ3/ɛ4 + ɛ4/ɛ4/2) (*r*_s_ = .47), and ɛ3/ɛ4 (*r*_s_ = .65) *vs* ɛ4/ɛ4 (*r*_s_ = .29)]. Results showed a lack of a ɛ4 general effect (*z* = .9; *p* = .168), as well as a gene-dose effect (*z* = 1.3; *p* = .170).

## 5 Discussion

In the current study, we explored the influence of the APOE genotype on learning contextual memories, orienting spatial attention based on those long-term memory traces, and their explicit recall. We found preserved long-term memory for object locations and identities within unique contexts in at-risk middle-aged individuals. Studies investigating the cognitive signature on memory of APOE genotype in middle-aged people, within a narrow age-range (40–50 years old) are scarce (for a recent review see Salvato, 2015). Greenwood and colleagues (2014) have shown no difference in memory in a middle-aged sample on a working-memory task. Moreover, Sierra-Fitzgerald, Barreto, and Lopera-Restrepo (2013) did not find any difference between middle-aged ɛ4-carriers and non-carriers using a wide range of neuropsychological tests. Evans et al. (2014) have found a better performance for ɛ4-carriers compared to non-carriers on a prospective memory task, but equal performance on a covert attention task.

Interestingly, we found a reliable LTM-based attention in the ɛ3/ɛ4 and ɛ4/ɛ4 groups. These results showed that our ɛ4-carrier participants were able to benefit from previous experience in orienting spatial attention. To our knowledge, this is the first study exploring the APOE genotype effect on LTM-based attention in middle-aged individuals. The only available evidence has reported a cognitive disadvantage for older ɛ4-carriers on a Contextual cueing (CC) task (Negash et al., 2007). In the CC paradigm the identification of a target is enhanced when it appears in a previously learnt location within repeated contextual configurations, optimizing perception for goal-directed behaviour (Chun and Jiang, 1998, Chun and Jiang, 2003). Negash et al. (2007) compared patients with Mild Cognitive Impairment to healthy controls. Additionally, the control group was separated into ɛ4-carriers (*n* = 11; mean age = 78.5; SD = 5.2) and non-carriers (*n* = 13; mean age = 74.5; SD = 4.5). The authors found an impaired contextual implicit learning in healthy older ɛ4 carriers, which showed the same pattern of results as of the MCI sample. The discrepancy between their results and ours may be accounted by the difference in age of the samples, as well as by the task used.

The most intriguing finding in our study was that the possession of the ɛ4 allele modulated the relationship between the explicit memory trace and the attention facilitation conferred by memory. In ɛ3/ɛ3 participants, the orienting effect was related to the explicit memory trace. This pattern mirrors what we have previously reported in younger healthy participants (Salvato et al., 2015). This correlation, however, dissipated with the possession of the ɛ4 allele. In ɛ3/ɛ4 participants, the orienting effect correlated with explicit memory for the object identity but not for the spatial location of the object. More interestingly, carrying two ɛ4 alleles disrupted those correlations further. However, comparing correlation coefficients between groups, we found a lack of gene-dose effect. These findings mimic what we previously observed in older participants (62–80 years old) (Salvato et al., 2015), and suggest that the memories guiding attentional orienting were not explicitly available to ɛ4/ɛ4 individuals.

Different explanations could account for these results. One possibility is that our findings point to a pattern of accelerated cognitive ageing in ɛ4/ɛ4 middle-aged individuals. Consistent with the notion of accelerated neural ageing, Evans et al. (2014) showed patterns of neural activity in middle-aged individuals similar to those usually observed in much older adults. These authors have observed decreased parietal and extrastriate activity in e4-carriers during prospective memory and attentional tasks, typically observed in healthy ageing (Evans et al., 2014). In the current study, our data possibly reflect a behavioural signature of precocious brain ageing. Middle-aged ɛ4 carriers and healthy older people presented an overlapping pattern of results, as in both cases the memory based orienting of attention did not depend on the quality declarative memory trace.

Another possibility relates to structural changes in the brain occurring earlier in middle-aged ɛ4-carriers. We have previously shown that a network of medial temporal, parietal, and frontal regions participates in memory-based orienting of attention, with a specific engagement of the hippocampus (Stokes et al., 2012, Summerfield et al., 2006). These brain areas are strongly compromised in neurodegenerative diseases (Braak & Braak, 1991). Interestingly, early anatomical and functional abnormalities in those regions have been reported in middle-aged ɛ4 individuals, though evidence is scarce. For instance, Reiman et al. (1996) in a PET study comparing ɛ4 homozygotes non-ɛ4 controls have shown a reduced glucose metabolism in the posterior cingulate, parietal, temporal, and prefrontal regions. Furthermore, Trivedi et al. (2006) found that ɛ3/ɛ4 compared to ɛ3/ɛ3 middle-aged individuals displayed reduced fMRI activation in the hippocampus and middle temporal lobe during an episodic encoding task, on which participants showed an equivalent behavioural performance. More recently, Trachtenberg et al. (2012) have shown a paradoxical activation of non task-related brain regions in ɛ4 carriers during a memory task, for which participants did not show any difference at the level of behavioural performance. Within this framework, early structural and functional changes in brain areas, subserving the LTM-based attention, might modulate the way explicit memory influences visuo-spatial attention in ɛ4-carriers. Our behavioural data could reflect compensatory mechanisms leading ɛ4 individuals to benefit from a memory store other then explicit ones, which contributes to a reliable memory-based orienting of attention.

Alternatively, a difference in learning might have contributed to our findings. The shallower learning slope in the ɛ4/ɛ4 group finds a possible parallel in the finding of a smaller neural investment required for learning by ɛ4/ɛ4 individuals (Mondadori et al., 2007). Mondadori and colleagues (2007) interpreted their findings as reflecting a more efficient use of memory resources in younger ɛ4-carriers. In our study, middle-aged ɛ4/ɛ4 showed a breakdown between declarative long-term memory and memory-based orienting although they were faster on the first learning block. Investigating rates of learning in different APOE groups over the lifespan may prove especially informative in revealing differential trajectories of learning and memory functions for the different genotypes, and exposing possible antagonistic pleiotropy (Tuminello & Han, 2011).

These findings may also reflect differences in our participants' learning style utilized to associate objects and scenes. Indeed, spatial or non-spatial approaches may have been used to learn the contextual associations. It has been demonstrated that spatial strategies are mainly hippocampus-dependent, while non-spatial strategies, such as relying on stimulus-response associations, are known to involve striatal systems (Bohbot et al., 2007, Iaria et al., 2003). In our case, these distinct learning strategies, involving distinct memory systems, may have differently contributed in orienting attention. However, we cannot explore strategy differences, as we did not collect any qualitative data on the encoding style of our participants.

This study had some limitations. Firstly, our groups may have been too small to detect an effect of APOE genotype on cognition. Furthermore, our participants were highly educated, and high level of education has been previously identified as a protective factor against dementia (Evans et al., 1997, Stern et al., 1994). Lastly, it would be useful to follow-up our participants' cognitive profile in order to detect critical behavioural changes in those who will suffer from neurodegenerative disease. Neuroimaging studies using sensitive tasks such as the one used in this study, which can tap into different types of associative memory with fine precision and measure their consequences on perception, may provide a promising avenue for investigating the influence of the APOE genotype the integrity of neural networks supporting cognition in middle-aged individuals.

## Figures and Tables

**Table 1:** Demographic characteristics of the final sample.

	*N*	Age	Education	Gender (M/F)	Family history (FH+/FH−)	Handedness (R/L)	ACE-III score
ɛ3/ɛ3	20	45.5 (±2.7)	16.1 (±.7)	9/11	5/15	18/2	96 (±.6)
ɛ3/ɛ4	20	46.3 (±3.1)	16.1 (±.6)	11/9	4/16	15/5	96.4 (±.7)
ɛ4/ɛ4	19	45.3 (±3.1)	16.4 (±.7)	6/13	4/15	17/2	96.6 (±.6)
